# Management Challenges and Outcomes of Bronchiolitis Obliterans Syndrome After Hematopoietic Stem Cell Transplantation: First Case Series Report From Vietnam

**DOI:** 10.1155/crh/8055215

**Published:** 2026-07-08

**Authors:** Luong Dinh-Van, Hoang-Anh Nguyen-Cong, Nguyen Tran-Ngoc, Bich-Ngoc T. Nguyen

**Affiliations:** ^1^ Lung Transplant Center, National Lung Hospital, Hanoi, Vietnam; ^2^ Center for Rare Lung Diseases and Respiratory Infections, National Lung Hospital, Hanoi, Vietnam; ^3^ Department of Tuberculosis and Lung Diseases, School of Medicine, University of Medicine and Pharmacy at Ho Chi Minh City, Ho Chi Minh, Vietnam, hcmut.edu.vn; ^4^ University of Medicine and Pharmacy, Vietnam National University, Hanoi, Vietnam, vnu.edu.vn

**Keywords:** bronchiolitis obliterans syndrome, corticosteroids, hematopoietic stem cell transplantation, invasive pulmonary aspergillosis, outcome

## Abstract

Bronchiolitis obliterans syndrome (BOS) is a serious pulmonary manifestation of chronic graft‐versus‐host disease (cGvHD) after allogeneic hematopoietic stem cell transplantation (HSCT). Data from low‐ and middle‐income countries remain scarce. We conducted a case series of 11 patients diagnosed with BOS between November 2023 and June 2025 at the National Lung Hospital, Hanoi, Vietnam. Clinical characteristics, treatments, pulmonary function, and outcomes over 12 months were analyzed. The series was predominantly male (81.8%), with a median age of 34 years (range, 12–57). The median time from HSCT to BOS diagnosis was 24 months (range, 11–72). All patients received fluticasone, azithromycin, montelukast (FAM) plus long‐acting bronchodilators, and 4 patients also received systemic corticosteroids. Median baseline forced expiratory volume in one second (FEV_1_) was 1.35 L (range, 0.53–2.48), corresponding to 40% predicted (range, 18–68). Over 12 months, 9/11 patients (81.8%) were hospitalized for respiratory infections, with invasive aspergillosis in 7/14 episodes (50%). Four patients (36.4%) died, three from infections, and one from autoimmune cerebritis. Median survival among deceased patients was 6.5 months. Among survivors, lung function was generally stable, with no significant change in FEV_1_ across baseline, 3, 6, and 12 months (Friedman test, *p* value = 0.37). BOS after HSCT in Vietnam was associated with high early mortality and a high incidence of invasive fungal disease. Systemic corticosteroid use appeared to increase infection‐related deaths, while steroid‐sparing regimens such as FAM combined with bronchodilators stabilized lung function in most survivors. Early detection, antifungal prophylaxis, and steroid‐sparing strategies are crucial in this high‐risk population.

## 1. Introduction

Following allogeneic hematopoietic stem cell transplantation (HSCT), patients may develop chronic graft‐versus‐host disease (cGvHD), of which bronchiolitis obliterans syndrome (BOS) represents a severe, noninfectious pulmonary manifestation. Clinical presentations such as dry cough, wheezing, and progressive dyspnea are due to narrowing of small airways, which limits airflow and ultimately leads to pulmonary function decline [1]. Although its exact mechanisms remain poorly understood, the pathogenesis of BOS is thought to involve conditioning chemotherapy, recurrent infections, microaspiration, and alloreactive immune responses. These factors trigger chronic inflammation and progressive remodeling of the small airways [2]. Despite its low incidence of approximately 3% to 6% following HSCT [3], BOS often carries a poor prognosis. Unlike many extrapulmonary manifestations of cGvHD, only about 20% of patients achieve a sustained clinical response [4]. BOS worsens clinical outcomes of patients after HSCT and could be fatal, with a 2–3‐year survival of 60%–75% and 5‐year survival of only 40%–50% [5]. The mainstay of therapy for symptomatic BOS or forced expiratory volume in one second (FEV_1_) below 80% is systemic corticosteroids and other immunosuppressants in refractory or nonresponsive cases [6]. However, given the substantial risk of infectious complications, a careful risk‐benefit assessment is imperative prior to treatment initiation. Triple therapy with inhaled fluticasone, azithromycin and montelukast (FAM) might be more efficient in stabilizing lung function with a safer profile [7]. To develop a better understanding of such rare disease in low‐resource settings, this case series was conducted to describe characteristics of patients with BOS and the treatment outcome over a period of 12 months.

## 2. Case Series

We included all patients (11 patients) with cGvHD who developed BOS and presented to Center for Rare Lung Diseases and Respiratory Infections, National Lung Hospital, Hanoi, Vietnam, from November 2023 to June 2025. Patients were diagnosed with BOS according to National Institutes of Health (NIH) diagnostic criteria in 2014 [8] with severity of cGvHD and BOS graded as mild, moderate, or severe. Demographic status, clinical information, radiographic findings, and results of pulmonary function tests were collected as well as treatment assessment over period of 12 months. Treatment outcomes were categorized as complete, partial, stable, or progressive disease according to NIH consensus for cGvHD [9]. Comparisons of paired FEV_1_ values between baseline and 12 months were performed using the Wilcoxon signed‐rank test, while changes across multiple time points (baseline, 3, 6, and 12 months) were analyzed using the Friedman test. A *p* value < 0.05 was considered statistically significant.

The series consisted of 11 patients, predominantly male (81.8%), with a median age of 34 years (range, 12–57). Most had hematological malignancies (81.8%), and all were allogeneic HSCT recipients. The median time from HSCT to BOS diagnosis was 24 months (range, 11–72). All patients presented with moderate to severe cGvHD, and the most common extrapulmonary involvements were ocular (81.8%) and oral (72.7%). The cardinal symptom was dyspnea on exertion, with patient No. 6 having dyspnea at rest. Air‐trapping on chest computed tomography and pulmonary function test was noticed in every patient. Median baseline FEV_1_ was 1.35 L (range, 0.53–2.48), corresponding to 40% predicted (range, 18–68); 4 patients (36.4%) had severe BOS (FEV_1_ < 40% predicted).

All patients received fluticasone, azithromycin, montelukast, and a long‐acting bronchodilator. Systemic corticosteroids (1 mg/kg prednisolone‐equivalent, tapered over 8 weeks) were given to 4 patients. Clinical features and treatments are summarized in Table 1.

**TABLE 1:** Summary of cases.

Case no	Age/sex	Underlying hematological disease	Post HSCT duration (months)	GvHD grade/management	WHO functional class	Baseline FEV1 liters (%)	BOS management	12‐month outcome/disease assessment
1	45/M	MDS	40	Severe (lung score: 2) steroid, ruxolitinib	III	1.27 (40)	FAM + LAB	Survival/stable
2	47/M	MF	36	Moderate (lung score: 1) ruxolitinib	II	2.14 (59)	FAM + LAB Steroids	Survival/stable
3	39/M	AA	23	Severe (lung score: 2, skin score: 3) CsA, ruxolitinib	II	1.77 (49)	FAM + LAB	Survival/stable
4	30/M	AML	24	Severe (lung score: 3) TAC, ruxolitinib	III	1.35 (35)	FAM + LAB Steroids	Death after 3 months
5	32/M	AML	12	Moderate (lung score: 1) steroid, TAC	II	2.48 (68)	FAM + LAB	Death after 12 months
6	24/M	Lymphoma	24	Severe (lung score: 3) steroid, TAC, ruxolitinib	IV	1.21 (25)	FAM + LAB Steroids	Death after 10 months
7	34/F	MDS	11	Severe (lung score: 3) steroid, TAC, ruxolitinib	III	0.53 (22)	FAM + LAB Steroids	Death after 1.5 months
8	42/M	ALL	11	Severe (lung score: 3) steroid, TAC	III	1.09 (33)	FAM + LAB	Survival/stable
9	52/M	AML	72	Moderate (lung score: 1) ruxolitinib	I	1.47 (67)	FAM + LAB	Survival/partial response
10	34/M	AML	24	Moderate (lung score: 1) steroid, TAC	II	2.39 (64)	FAM + LAB	Survival/stable
11	12/M	AA	24	Severe (lung score: 2) CsA	II	0.85 (40)	FAM + LAB	Survival/stable

*Note:* CsA, cyclosporine A; F, female; M, male; MDS, myelodysplastic syndrome; MF, myelofibrosis; TAC, tacrolimus.

Abbreviations: AA, aplastic anemia; ALL, acute lymphoblastic leukemia; AML, acute myeloid leukemia; FAM, fluticasone, azithromycin, and montelukast; LAB, long‐acting bronchodilator; WHO, World Health Organization.

During 12 months, 9/11 patients (81.8%) experienced ≥ 1 hospitalization for respiratory infections, with 14 episodes recorded. Invasive aspergillosis accounted for 7/14 episodes (50%).

**FIGURE 1:**
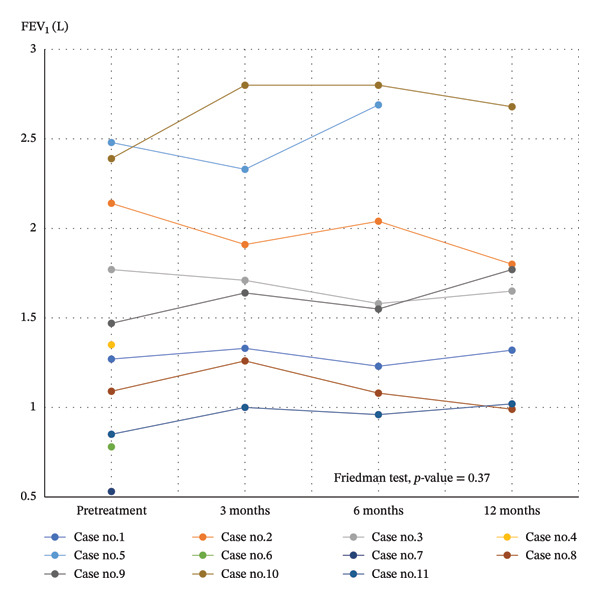
FEV1 assessment over 12 months.

At the end of study, 4 patients (36.4%) had died: 3 from severe infections and 1 from autoimmune cerebritis. Median survival among deceased patients was 6.5 months (range, 1.5–12) after BOS diagnosis. Among survivors, pulmonary function was generally stable, with one partial response. Median baseline FEV_1_ did not differ significantly between survivors and nonsurvivors (1.47 L [range, 0.85–2.39]; 49% predicted [range, 33–67] vs. 1.07 L [range, 0.53–2.48]; 28.5% predicted [range, 18–68]; *p* value = 0.45 and *p* value = 0.25, respectively). In surviving patients, median FEV_1_ at 12 months was 1.65 L (range, 0.99–2.68; 48% predicted, range 30–88), not significantly different from baseline (*p* value = 0.74 and *p* value = 0.27, respectively). No overall change across baseline, 3, 6, and 12 months was detected (Friedman test, *p* value = 0.37) (Figure 1).

## 3. Discussion

BOS remains one of the most severe pulmonary manifestations of cGvHD after allogeneic HSCT. In our case series, the 12‐month mortality rate reached 36.4%, with the majority of deaths attributable to infectious complications. These findings emphasize the dual challenge of controlling BOS progression while simultaneously mitigating the risk of life‐threatening infections in highly immunocompromised patients.

The survival outcome in our series is broadly consistent with prior international studies, although the time frame differs. Large cohorts from the United States and Europe report 2–5‐year survival rates of 40%–60% following BOS diagnosis [10]. Asian studies have shown similarly poor outcomes: In a Korean cohort, the 3‐year survival of BOS patients was below 50%, and low baseline FEV_1_ strongly predicted mortality [11]. Our 12‐month mortality rate, therefore, appears high in comparison, reflecting both the severity of lung function impairment at diagnosis and the burden of infection in our setting.

Notably, we observed a high rate of respiratory infections. More than 80% of our patients required hospitalization for pulmonary infections, and invasive aspergillosis accounted for half of these events. This contrasts with published data showing cumulative invasive fungal infection rates of approximately 7%–16% at 1 year after allo‐HSCT [12]. The discrepancy may be explained by several factors: the referral nature of our center, where patients already had advanced chronic GvHD and prolonged immunosuppression; the absence of universal antifungal prophylaxis during BOS treatment; and the intensive fungal screening program at our institution, which may have increased diagnostic yield. This high incidence of fungal disease highlights the importance of early prophylaxis and vigilant surveillance in this population.

The relationship between systemic corticosteroid use and infectious outcomes was particularly evident in our series. Three of the four patients who received systemic steroids died from severe infections within the first year. This observation aligns with international experience: Prolonged systemic corticosteroid courses are no longer recommended as first‐line therapy for BOS, as they are associated with increased risks of serious opportunistic infections and infection‐related mortality [13]. Although high‐dose steroids have been trialed, reported benefits are modest and often outweighed by infectious toxicity. In contrast, inhaled corticosteroid‐based strategies (such as budesonide/formoterol or the FAM regimen) have demonstrated the ability to stabilize lung function with a safer infectious profile [14]. Our data support minimizing systemic steroid exposure and adopting steroid‐sparing regimens whenever possible.

Another important lesson from our series is the value of early diagnosis and intervention. Most of our patients were diagnosed late, with already advanced airflow obstruction (median baseline FEV_1_ 1.35 L, with four cases having FEV_1_ below 40% predicted). Previous studies suggest that earlier initiation of FAM therapy, even before overt obstruction, may slow disease progression [7]. Unfortunately, pulmonary function tests are not routinely performed during post‐HSCT surveillance in many centers, leading to delayed recognition of BOS. Integrating regular spirometry or home‐based pulmonary monitoring may allow earlier detection and therapeutic intervention in the future.

While early detection is paramount, optimizing the subsequent pharmacological management remains a major clinical challenge. In our series, 8 of the 11 patients received calcineurin inhibitors (CNIs) as part of their systemic cGvHD management. For decades, CNIs have served as a cornerstone steroid‐sparing therapy for cGvHD and its pulmonary manifestations [15]. The use of CNIs to manage BOS is further supported by its ability to dampen immune‐mediated fibrotic cascades. Similar fibrotic mechanisms, driven by epithelial‐mesenchymal transition and profibrotic cytokines, are seen in autoimmune disorders like systemic sclerosis, where long‐term CNI therapy has been shown to stabilize progressive lung fibrosis [16, 17]. However, in both transplant and autoimmune settings, the overall clinical evidence for long‐term antifibrotic success is still weak, and patient outcomes remain highly variable. Given this unpredictability, personalized treatment is essential. Pharmacogenomics is already utilized in other immune‐mediated diseases to guide therapy. As highlighted by Murdaca et al. in the context of autoimmune diseases, pharmacogenomic profiling permits the tailored administration of biological agents; for instance, specific tumor necrosis factor‐alpha polymorphisms (such as the −308 G/G or +489 GG genotypes) can effectively predict a favorable therapeutic response to etanercept in patients with psoriatic arthritis [18, 19]. Applying this pharmacogenomic approach to the post‐allo‐HSCT setting could help optimize standard immunosuppressive regimens. Genetic variations in drug‐metabolizing enzymes and transporters, such as cytochrome P450 (CYP) 3A5 and ATP‐binding cassette transporter family polymorphisms, have been shown to significantly alter the pharmacokinetics of CNIs like tacrolimus and cyclosporine, ultimately influencing GvHD outcomes and toxicity [20, 21]. This concept is especially relevant for our series, where the majority of patients (7/11) received the Janus kinase (JAK) 1/2 inhibitor ruxolitinib. Ruxolitinib is predominantly metabolized by CYP3A4, and its concurrent use with strong CYP3A4 inhibitors, such as posaconazole, which is frequently required for invasive fungal prophylaxis, can lead to significant drug–drug interactions and ruxolitinib toxicity [22]. Integrating pharmacogenomic screening could help clinicians anticipate individual metabolic capacities, thereby optimizing the dosing of targeted therapies and CNIs. This individualized approach could help achieve effective BOS control while minimizing the risk of severe opportunistic infections.

Our study has limitations, primarily the small sample size and single‐center design. Selection bias is inevitable, as all cases were referrals with moderate to severe chronic GvHD, which may overestimate mortality and infection rates compared with unselected HSCT populations. Nevertheless, this is, to our knowledge, the first Vietnamese case series of BOS after HSCT, providing unique real‐world data from a resource‐limited setting.

In conclusion, BOS after HSCT is a severe complication with high mortality and morbidity. Our findings highlight that systemic corticosteroids carry a considerable infection‐related risk, particularly invasive aspergillosis, and should be reserved for carefully selected patients with rapidly progressive disease. Steroid‐sparing regimens such as FAM combined with long‐acting bronchodilators appear safer and may stabilize lung function in most survivors. Future studies should focus on strategies for earlier diagnosis, standardized antifungal prophylaxis, and integration of targeted immunomodulatory therapies to improve outcomes in this vulnerable population.

**Nomenclature**
BOS: Bronchiolitis obliterans syndrome
cGvHD: Chronic graft‐versus‐host disease
CNIs: Calcineurin inhibitors
CYP: Cytochrome P450
FAM: Fluticasone, azithromycin, montelukast
FEV_1_
: Forced expiratory volume in one second
HSCT: Hematopoietic stem cell transplantation
JAK: Janus kinase
NIH: National Institutes of Health

## Author Contributions

Luong Dinh‐Van and Hoang‐Anh Nguyen‐Cong contributed to literature review and drafting of the manuscript. Nguyen Tran‐Ngoc contributed to the review and editing of the manuscript. Bich‐Ngoc T. Nguyen supervised the project. Bich‐Ngoc T. Nguyen had full access to all of the data in this study and takes complete responsibility for the integrity of the data and the accuracy of the data analysis.

## Funding

The authors have nothing to report.

## Disclosure

All authors have read and approved the final version of the manuscript.

## Ethics Statement

The authors declare that written informed consent was obtained from all the patients for the publication of this manuscript and accompanying images and attest that the form used to obtain consent from the patients complies with the Journal requirements as outlined in the author guidelines. Institutional ethical approval was waived given the retrospective observational nature of this case series, in accordance with local regulations.

## Conflicts of Interest

The authors declare no conflicts of interest.

## Data Availability

Anonymized data can be made available upon request. Requests should be addressed to Bich‐Ngoc T. Nguyen (ngocn4@hotmail.com).
